# The association between high atherogenic index of plasma and impaired lung function: a population-based study

**DOI:** 10.3389/fmed.2025.1589605

**Published:** 2025-07-09

**Authors:** Liang Yang, Yuanzhou Wu, Ling Chen, Zizhao Li, Wenfei Zhu, Ziyan Zhang, Hui Li, Yang Huang, Qunqing Chen

**Affiliations:** Department of Thoracic Surgery, Zhujiang Hospital, Southern Medical University, Guangzhou, China

**Keywords:** atherogenic index of plasma, NHANES, lung function, cross-sectional study, non-linear association

## Abstract

**Objective:**

Although AIP is a recognized cardiovascular risk marker, its association with pulmonary function and sex-specific differences remains unclear. This study investigated whether elevated AIP is independently associated with reduced lung function and examined potential sex-specific patterns.

**Methods:**

Data from 4,565 participants in the NHANES 2007–2012 dataset were analyzed using a cross-sectional design. AIP served as the exposure variable, with five lung function metrics (including FEV_1_, FVC, and FEV_1_/FVC ratio) as outcomes. Weighted multiple linear regression, threshold effect analysis, subgroup comparisons, and XGBoost modeling were performed to assess associations.

**Results:**

Multivariable regression showed a significant negative association between AIP and FEV_1_ (β = −121.3 mL/unit, *p* < 0.001) and FVC (β = −147.1 mL/unit, *p* < 0.001), with no significant link to FEV_1_/FVC ratio. Subgroup analysis revealed a U-shaped non-linear association in females, with inflection points at AIP values of 0.77 (FEV_1_) and 0.78 (FVC), beyond which declines in lung function plateaued. Males exhibited a consistent negative correlation across all AIP levels.

**Conclusion:**

Elevated AIP is independently associated with reduced lung function, particularly non-linear effects in females. These findings support AIP as a potential adjunct marker for pulmonary function assessment in clinical practice.

## 1 Introduction

Spirometry-based parameters, such as forced expiratory volume in 1 s (FEV_1_), forced vital capacity (FVC), and peak expiratory flow (PEF), serve as fundamental metrics for evaluating respiratory function and tracking disease progression (1, 2). Beyond respiratory diseases, reduced pulmonary function has also been associated with elevated risks of perioperative complications, cardiovascular conditions, insulin resistance, and overall mortality (3–5). Various investigations have proposed that systemic inflammation and vascular remodeling may underlie these associations, establishing a link between impaired lung function and subclinical atherosclerosis as well as metabolic dysfunction (6–9).

The atherogenic index of plasma (AIP), defined as the logarithmic ratio of triglycerides to high-density lipoprotein cholesterol (log [TG/HDL-C]), is a comprehensive indicator of lipid metabolism that encompasses both atherogenic propensity and systemic inflammation (10). AIP has demonstrated superior predictive value for cardiovascular disease compared to traditional lipid markers and has been increasingly utilized as a proxy for metabolic risk in clinical and epidemiological investigations (11–13). Moreover, lipid abnormalities and chronic low-grade inflammation have been implicated in pulmonary dysfunction, suggesting a potential association between lipid profiles and lung health (14–17). Nevertheless, despite the growing recognition of AIP as a cardiovascular risk marker, its relationship with pulmonary function remains underexplored, particularly in large-scale, population-based studies. Previous research has typically focused on individual lipid components rather than integrated indices such as AIP, which could potentially offer a more comprehensive depiction of the metabolic-inflammatory status pertinent to lung function.

To address this gap, the present study employed data from the National Health and Nutrition Examination Survey (NHANES) 2007–2012 to investigate the association between AIP and spirometry-based lung function in a nationally representative adult population (18). Understanding this relationship could enhance the early detection of individuals vulnerable to metabolic-associated pulmonary dysfunction and enhance comprehensive risk assessment approaches in clinical settings.

## 2 Materials and methods

### 2.1 Study participants

NHANES represents a nationally inclusive survey of the U.S. populace conducted by the CDC through a complex, multistage probability sampling design. The current investigation utilized a cross-sectional design using NHANES data, which were collected at a single time point without longitudinal tracking. For each survey cycle, participants are selected through stratified sampling based on geographic and demographic variables. All procedures are approved by the National Center for Health Statistics Research Ethics Review Board, and informed consent is secured from all participants.

**FIGURE 1:**
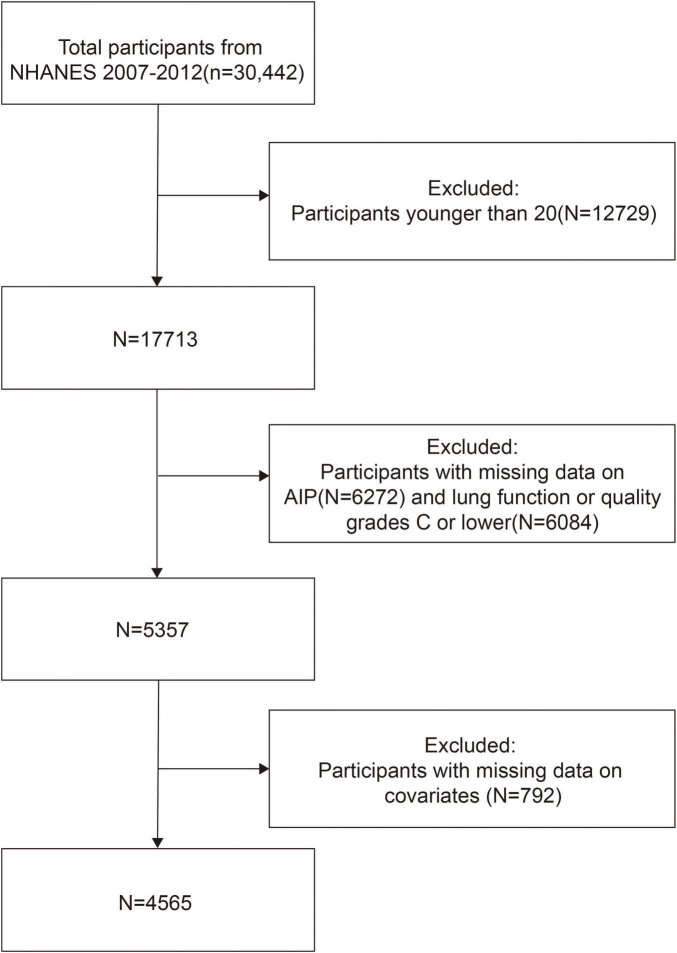
Flowchart of the sample selection in this study.

In this study, we included data from the 2007 to 2012 cycles. Participants under 20 years of age (*n* = 12,729) were excluded to focus on the adult population. We also excluded individuals with missing AIP data (*n* = 6,272), those with incomplete or poor-quality spirometry data (*n* = 6,084), and those with missing covariates (*n* = 792), resulting in a final analytic sample of 4,565 participants. The sample selection process is illustrated in Figure 1.

### 2.2 Study variables

#### 2.2.1 AIP

AIP is an exposure metric derived from the formula log [TG (mg/dL)/HDL-C (mg/dL)] (10, 19, 20).

#### 2.2.2 Lung function assessment

To assess pulmonary function, five spirometry-derived indices—FEV_1_, FVC, PEF, FEF25–75%, and FEV_1_/FVCd—PEFestablished as outcome variables. Every employee at NHANES has learnt spirometry, which was developed in accordance with the protocols set out by the American Thoracic Society (ATS). Five letters were used to score the spirometry results for each technician: A, B, C, D, and F. Only data that met or exceeded the ATS/ERS criteria, as determined by FEV_1_ and FVC quality ratings of A and B, were utilized in the study to ensure data quality (21).

### 2.3 Selection of Covariates

The supplementary variables were selected based on previous studies concerning pulmonary function: Gender, age, ethnicity, educational level, body mass index, total cholesterol, smoking habits, alcohol intake, hypertension, diabetes, and serum cotinine concentrations. These variables are described and categorized in depth in Supplementary Table S1.

### 2.4 Statistical analysis

In order to account for the intricate, multistage architecture of the NHANES survey, the research followed CDC recommendations and used suitable weighting techniques. The supplementary variables were selected based on previous studies concerning lung function: Gender, age, ethnicity, educational level, body mass index, total cholesterol, smoking habits, alcohol intake, hypertension, diabetes, and serum cotinine concentrations. Using these three weighted regression models, the relationship between AIP and lung function was examined. Subgroup analyses were used to investigate the disparities among different covariate groupings. AIP and lung function were examined for potential non-linear connections using threshold effect analysis and smoothed curve fitting. To assess the robustness of the findings, sensitivity analyses were conducted by excluding participants with self-reported diabetes or hypertension. Additionally, an XGBoost regression model was constructed to evaluate the relative importance of lipid-related indicators in predicting pulmonary function. The dataset was randomly divided into a training set (80%) and a test set (20%) using stratified sampling. Hyperparameters such as learning rate, maximum tree depth, and number of estimators were tuned via grid search with fivefold cross-validation. Early stopping and L2 regularization were applied to prevent overfitting. Model performance was assessed using the root mean squared error (RMSE) on the test set. To interpret the model output, SHAP (Shapley Additive Explanations) values were calculated to quantify the contribution of each feature. All analyses were implemented using the xgboost, shapviz, and kernelshap packages in R. For all statistical analyses, R version 4.2.3^1^ and Empower Stats^2^ were used. *P*-values below 0.05 were considered statistically significant.

## 3 Results

### 3.1 Baseline characteristics

The average age for the 4,565 individuals was 46.56 ± 16.19 years, with 2,268 males (49.68%) and 2,297 females (50.32%). FEV_1_: 3,089.72 ± 887.92 mL, FVC: 3,956.87 ± 1,075.87 mL, PEF: 8,123.37 ± 2,186.56 mL, FEF25–75%: 2,913.68 ± 1,283.88 mL, and the ratio of FEV_1_/FVC: 0.78 ± 0.08 were the average lung function indices. On the basis of AIP, participants were divided into quartiles. There were significant differences (all *P* < 0.05) in age, gender, race, education level, smoking status, diabetes, hypertension, BMI, serum cotinine, total cholesterol, FVC, FEV_1_/FVC, and PEF among participants in the various AIP quartile groups (Table 1).

**TABLE 1:** Weighted baseline characteristics of the study population according to AIP.

	AIP Q1	AIP Q2	AIP Q3	AIP Q4	*P*-value
	(–0.79–0.11)	(0.11–0.32)	(0.32–0.54)	(0.54–2.06)	
Age (years)	43.67 ± 15.27	44.26 ± 15.38	46.47 ± 15.82	47.01 ± 14.10	< 0.001
Sex, n(%)					< 0.001
Male	371 (32.52%)	524 (45.94%)	620 (54.37%)	727 (63.57%)	
Female	770 (67.48%)	616 (54.06%)	520 (45.63%)	417 (36.43%)	
Race/ethnicity, n(%)					< 0.001
Mexican American	54 (4.70%)	85 (7.45%)	99 (8.69%)	114 (9.96%)	
Other Hispanic	47 (4.16%)	54 (4.70%)	64 (5.65%)	63 (5.47%)	
Non-Hispanic White	811 (71.07%)	839 (73.58%)	818 (71.72%)	845 (73.85%)	
Non-Hispanic Black	167 (14.67%)	109 (9.56%)	83 (7.24%)	57 (4.96%)	
Other races	61 (5.39%)	54 (4.72%)	76 (6.71%)	66 (5.77%)	
Education level, n(%)					< 0.001
Less than high school	105 (9.23%)	174 (15.25%)	190 (16.71%)	220 (19.27%)	
High school or GED	189 (16.57%)	241 (21.11%)	236 (20.73%)	279 (24.40%)	
Above high school	847 (74.20%)	725 (63.64%)	713 (62.56%)	644 (56.33%)	
Income to poverty ratio, n(%)					0.565
<1	141 (12.33%)	147 (12.88%)	140 (12.24%)	161 (14.03%)	
≥ 1	1,000 (87.67%)	993 (87.12%)	1,000 (87.76%)	983 (85.97%)	
Alcohol intake, n (%)					0.292
Yes	928 (81.31%)	906 (79.45%)	927 (81.30%)	945 (82.58%)	
No	213 (18.69%)	234 (20.55%)	213 (18.70%)	199 (17.42%)	
Smoke status, n (%)					< 0.001
Yes	417 (36.56%)	487 (42.70%)	542 (47.53%)	601 (52.55%)	
No	724 (63.44%)	653 (57.30%)	598 (52.47%)	543 (47.45%)	
Diabetes, n (%)					< 0.001
Yes	41 (3.58%)	58 (5.11%)	111 (9.72%)	145 (12.68%)	
No	1,100 (96.42%)	1,082 (94.89%)	1,029 (90.28%)	999 (87.32%)	
Hypertension, n(%)					< 0.001
Yes	231 (20.25%)	303 (26.56%)	381 (33.43%)	463 (40.46%)	
No	910 (79.75%)	837 (73.44%)	759 (66.57%)	681 (59.54%)	
BMI (kg/m^2^)					< 0.001
<25(kg/m^2^)	609 (53.37%)	394 (34.56%)	264 (23.18%)	132 (11.58%)	
25–29.9(kg/m^2^)	336 (29.42%)	414 (36.32%)	409 (35.87%)	420 (36.68%)	
≥ 30(kg/m^2^)	196 (17.21%)	332 (29.11%)	467 (40.95%)	592 (51.74%)	
Cotinine(ng/mL)	35.67 ± 96.48	52.31 ± 121.54	59.51 ± 127.92	75.03 ± 145.91	0.105
Total cholesterol (mg/dL)	188.56 ± 36.20	192.22 ± 38.05	195.58 ± 41.02	206.26 ± 43.99	< 0.001
Lung function					
FEV_1_(mL)	3179.10 ± 873.46	3262.17 ± 885.12	3250.54 ± 895.49	3244.30 ± 878.21	0.105
FVC (mL)	4051.15 ± 1055.17	4186.88 ± 1078.79	4190.28 ± 1083.45	4210.96 ± 1084.80	< 0.001
FEV_1_/FVC (%)	0.78 ± 0.08	0.78 ± 0.08	0.78 ± 0.08	0.77 ± 0.07	< 0.001
PEF (mL/s)	8138.27 ± 2071.84	8373.00 ± 2131.47	8441.67 ± 2192.76	8572.65 ± 2175.37	< 0.001
FEF25–75% (mL/s)	2967.54 ± 1233.48	3046.15 ± 1311.97	3020.24 ± 1292.72	2945.40 ± 1232.34	0.205

Continuous variables are expressed as mean ± SD, with *P*-values calculated using a weighted linear regression model. Categorical variables are expressed as percentages (%), and *P*-values were calculated using a weighted chi-square test.

### 3.2 AIP’s association with lung function

Significant association between AIP and the three lung function indicators of FEV_1_, FVC, and FEF25–75% were found using weighted multiple linear regression analysis (Table 2). In Model 3, the completely modified model, AIP exhibited a negative association with FEV_1_ (β = –121.3, 95% CI: –176.3, –66.2). The negative association remained evident when AIP was stratified into quartiles, exhibiting a β of –109.74 (95% CI: –158.58, –60.89) in the highest quartile (Q4), with the effect size amplifying with increasing AIP levels (P trend < 0.001). Comparable patterns were reported for FVC and FEF25–75%. Nonetheless, AIP revealed no significant connection with PEF (*P* = 0.084) or FEV_1_/FVC (*P* = 0.703).

**TABLE 2:** AIP’s association with lung function.

Lung function	AIP	Model 1		Model 2		Model 3	
		β (95%CI)	*P*-value	β (95%CI)	*P*-value	β (95%CI)	*P*-value
**FEV_1_(mL)**							
	Continuous	76.0 (–4.6, 156.6)	0.065	–207.4 (–258.3, –156.5)	< 0.001	–121.3 (–176.3, –66.2)	< 0.001
	Q1(–0.79–0.11)	Reference(0)		Reference(0)		Reference(0)	
	Q2(0.11–0.32)	83.07 (10.96, 155.19)	0.024	–59.19 (–103.22, –15.17)	0.008	–25.86 (–69.23, 17.51)	0.243
	Q3(0.32–0.54)	71.44 (–1.00, 143.89)	0.053	–88.30 (–133.12, –43.48)	<0.001	–33.26 (–78.67, 12.14)	0.151
	Q4(0.54–2.06)	65.20 (–7.75, 138.15)	0.080	–186.01 (–231.87, –140.14)	<0.001	–109.74 (–158.58, –60.89)	<0.001
	p for trend	0.119		<0.001		<0.001	
**FVC(mL)**							
	Continuous	179.9 (81.7, 278.1)	< 0.001	–271.0 (–333.8, –208.2)	< 0.001	–147.1 (–215.0, –79.2)	< 0.001
	Q1(–0.79–0.11)	Reference(0)		Reference(0)		Reference(0)	
	Q2(0.11–0.32)	135.72 (47.90, 223.55)	0.003	–68.80 (–123.13, –14.48)	0.013	–22.67 (–76.19, 30.85)	0.406
	Q3(0.32–0.54)	139.13 (50.89, 227.36)	0.002	–118.83 (–174.13, –63.53)	<0.001	–40.89 (–96.92, 15.14)	0.153
	Q4(0.54–2.06)	159.80 (70.96, 248.65)	<0.001	–233.47 (–290.06, –176.88)	<0.001	–126.34 (–186.62, –66.06)	<0.001
	p for trend	<0.001		<0.001		<0.001	
**FEV_1_/FVC**							
	Continuous	0.0 (0.0, 0.0)	< 0.001	0.00 (0.00, 0.00)	0.607	0.00 (0.00, 0.00)	0.703
	Q1(–0.79–0.11)	Reference(0)		Reference(0)		Reference(0)	
	Q2(0.11–0.32)	–0.01 (–0.01, 0.00)	0.1071	0.00 (–0.01, 0.00)	0.682	0.00 (–0.01, 0.00)	0.486
	Q3(0.32–0.54)	–0.01 (–0.01, 0.00)	0.009	0.00 (0.00, 0.01)	0.615	0.00 (–0.01, 0.01)	0.951
	Q4(0.54–2.06)	–0.01 (–0.02, –0.01)	<0.001	0.00 (–0.01, 0.01)	0.915	0.00 (–0.01, 0.00)	0.427
	p for trend	<0.001		0.891		0.547	
**PEF (ml/s)**							
	Continuous	579.0 (383.6, 774.4)	<0.001	–268.0 (–414.9, –121.0)	<0.001	–137.8 (–294.3, 18.7)	0.084
	Q1(–0.79–0.11)	Reference(0)		Reference(0)		Reference(0)	
	Q2(0.11–0.32)	234.73 (59.73, 409.72)	0.009	–127.44 (–254.37, –0.52)	0.049	–74.99 (–198.32, 48.35)	0.234
	Q3(0.32–0.54)	303.40 (127.59, 479.20)	<0.001	–190.81 (–320.01, –61.60)	0.004	–117.95 (–247.08, 11.17)	0.074
	Q4(0.54–2.06)	434.38 (257.35, 611.41)	<0.001	–303.09 (–435.31, –170.87)	<0.001	–199.49 (–338.42, –60.57)	0.005
	p for trend	<0.001		<0.001		0.004	
**FEF25–75%(mL/s)**							
	Continuous	–27.4 (–143.2, 88.4)	0.643	–129.9 (–221.3, –38.5)	0.005	–152.0 (–251.8, –52.2)	0.003
	Q1(–0.79–0.11)	Reference(0)		Reference(0)		Reference(0)	
	Q2(0.11–0.32)	78.61 (–24.97, 182.20)	0.137	–10.60 (–89.59, 68.38)	0.793	–12.79 (–91.44, 65.86)	0.750
	Q3(0.32–0.54)	52.70 (–51.36, 156.77)	0.321	2.21 (–78.20, 82.62)	0.957	–2.77 (–85.12, 79.57)	0.947
	Q4(0.54–2.06)	–22.14 (–126.93, 82.65)	0.679	–123.20 (–205.48, –40.91)	0.003	–133.44 (–222.03, –44.85)	0.003
	p for trend	0.564		0.005		0.005	

Model 1 was not modified. Age, sex, and race were adjusted for Model 2. Every variable in Model 3 has been adjusted.

### 3.3 Subgroup analyses

**FIGURE 2:**
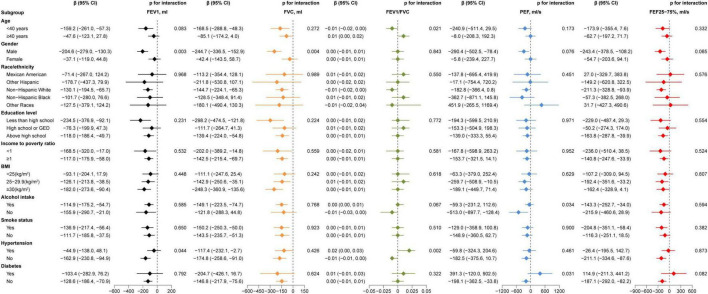
Subgroup analyses between AIP and lung function. Adjusted for all variables.

In order to examine the connections between AIP and lung function in different subgroups, we performed subgroup analysis. Gender affected the association between AIP and FEV_1_ (*P* = 0.003) and FVC (*P* = 0.004), different age affected the association AIP and FEV_1_/FVC (*P* = 0.021), as Figure 2. Furthermore, the link between AIP and PEF was impacted by alcohol use (*P* = 0.034) and diabetes status (*P* = 0.031), whilst the association between AIP and the FEV_1_/FVC (*P* = 0.002) and the FEV_1_ (*P* = 0.044) were modified by the presence of hypertension. Figure 2 provides further information.

### 3.4 Threshold effect analysis

**FIGURE 3:**
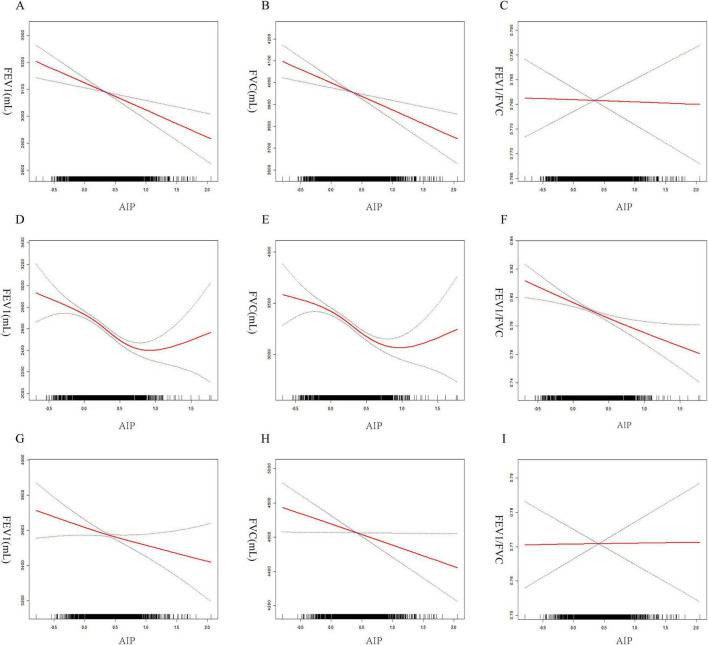
Smoothed curve fitting between AIP and lung function. All participants **(A)** AIP and FEV_1_, **(B)** AIP and FVC, **(C)** AIP and FEV_1_/FVC. Females **(D)** AIP and FEV_1_, **(E)** AIP and FVC, **(F)** AIP and FEV_1_/FVC. Males **(G)** AIP and FEV_1_, **(H)** AIP and FVC, **(I)** AIP and FEV_1_/FVC.

To ensure the reliability of the regression analysis results, the impact of AIP on pulmonary function was also investigated using threshold effect analysis and smooth curve fitting. The results continuously showed a negative relationship between the two (Figures 3A–C). According to gender-stratified study, there may be a U-shaped relationship between female lung function and AIP (Figures 3D–F). In contrast, males appeared to consistently exhibit a negative association between AIP and pulmonary function (Figures 3G–I). Threshold effect study revealed that, for AIP < 0.77, AIP had a negative correlation with FEV_1_ in females (β = –147.6, 95% CI: –228.4, –66.8). Nevertheless, beyond the inflection point, this favorable connection ceased to be substantial (Table 3). In females, there was a same pattern between AIP and FVC. Males, on the other hand, consistently showed a negative correlation between AIP and lung function.

**TABLE 3:** Threshold effect analysis of AIP and lung function.

Lung function		Adjusted β(95%CI) *P*-value	
		Female	Male
**FEV_1_(mL)**			
	Inflexion point	0.77	0.23
	AIP < K effect	–147.6 (–228.4, –66.8) <0.001	–370.6 (–656.6, –84.6) 0.011
	AIP > K effect	338.9 (–27.9, 705.8) 0.070	46.4 (–68.5, 161.4) 0.429
	*P* for log-likelihood ratio	0.015	0.013
**FVC (mL)**			
	Inflexion point	0.78	0.22
	AIP < K effect	–159.7 (–255.2, –64.2) 0.001	–395.5 (–732.9, –58.1) 0.022
	AIP > K effect	286.7 (–146.9, 720.2) 0.195	4.3 (–131.3, 139.9) 0.950
	*P* for log-likelihood ratio	0.048	0.051
**FEV_1_/FVC**			
	Inflexion point	0.67	–0.06
	AIP < K effect	0.0 (0.0, 0.0) 0.459	–0.1 (–0.2, 0.0) 0.038
	AIP > K effect	0.0 (0.0, 0.1) 0.243	0.0 (0.0, 0.0) 0.041
	*P* for log-likelihood ratio	0.213	0.019

Every variable was adjusted.

### 3.5 Analysis of important lipid variables based on XGBoost machine learning

**FIGURE 4:**
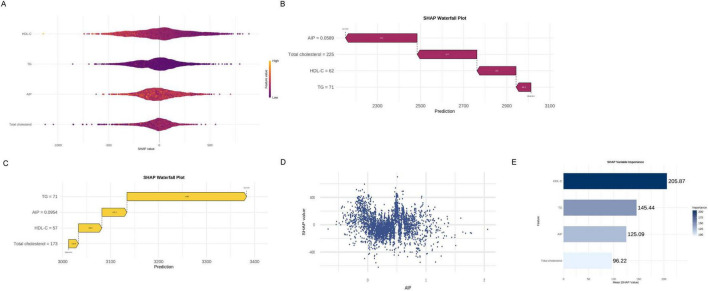
Analysis of important lipid variables based on XGBoost machine learning. **(A)** SHAP beeswarm plot. **(B)** SHAP waterfall plot (low FEV_1_ case). **(C)** SHAP waterfall plot (high FEV_1_ case). **(D)** SHAP dependence plot of AIP. **(E)** SHAP summary bar plot.

To explore the relationship between lipid metabolism and pulmonary function, we constructed an XGBoost regression model with forced expiratory volume in 1 s (FEV_1_) as the outcome. Four pre-defined lipid-related indicators were included: the atherogenic index of plasma (AIP), triglycerides (TG), high-density lipoprotein cholesterol (HDL-C), and total cholesterol. Model interpretation using SHapley Additive exPlanations (SHAP) enabled evaluation of the direction and magnitude of each variableach variableh varAs shown in the SHAP beeswarm plot (Figure 4A), higher AIP values were consistently associated with lower predicted FEV_1_, indicating a negative association. In one representative case (Figure 4B), an individual with an AIP value of 0.0589 exhibited a SHAP value of –333 for AIP, representing the largest single-variable contribution to reduced FEV_1_. Conversely, another individual with a higher AIP value of 0.0954 showed a positive SHAP value of + 52.3 (Figure 4C), suggesting a potentially non-linear or threshold-dependent association. The SHAP dependence plot of AIP (Figure 4D) further demonstrated a dispersed, non-monotonic pattern, highlighting individual heterogeneity in its relationship with predicted lung function. In the SHAP summary bar plot (Figure 4E), HDL-C exhibited the highest average contribution to the model output, followed by TG and AIP, while total cholesterol had a comparatively smaller effect. These findings highlight the potential relevance of AIP as an indicator associated with reduced pulmonary function, while also emphasizing its context-specific impact. Further longitudinal research is warranted to evaluate its clinical utility.

### 3.6 Sensitivity analysis

To assess the robustness of the findings, a sensitivity analysis was conducted by excluding 1,733 participants with self-reported diabetes or hypertension. In Model 2 (adjusted for age, sex, and race), AIP remained significantly and negatively associated with all five lung function parameters: FEV_1_, FVC, FEV_1_/FVC, PEF, and FEF25–75%.In the fully adjusted Model 3, these associations persisted and became more pronounced: FEV_1_ (β = –168.77, 95% CI: –238.44, –99.09), FVC (β = –171.89, 95% CI: –259.63, –84.15), FEV_1_/FVC (β = –0.01, 95% CI: –0.02, –0.00), PEF (β = –227.76, 95% CI: –424.50, –31.03), and FEF25–75% (β = –238.10, 95% CI: –367.91, –108.29). These results confirm that the inverse association between AIP and pulmonary function is robust and not driven by participants with major chronic conditions (see Supplementary Table S2).

## 4 Discussion

This study analyzed NHANES data from 2007 to 2012 and found a consistent inverse association between the AIP and pulmonary function in U.S. adults, independent of multiple confounders. Through multivariate regression analysis, it was determined that each incremental unit rise in AIP corresponded to a reduction of 121.3 mL in FEV_1_ and 147.1 mL in FVC, with no significant association observed for FEV_1_/FVC. These findings suggest that heightened AIP levels are associated with a restrictive pattern of lung impairment rather than an obstructive one, aligning with prior investigations on metabolic syndrome and pulmonary function (22, 23). Subsequent subgroup analyses demonstrated significant variations in this association based on diabetes status, hypertension, and gender. Additionally, XGBoost machine learning techniques identified AIP as a key lipid-related predictor of lung function.

Although the association between metabolic syndrome and impaired lung function is well established, limited research has focused on the specific role of AIP. Leone et al. reported that reduced HDL-C and elevated triglycerides were strongly linked to lower FEV_1_ and FVC, with abdominal obesity emerging as the most predictive factor (17). Similar trends were confirmed in Asian cohorts (24) and the EpiHealth study (25), revealing that central adiposity, more so than BMI, was closely linked to declines in lung function. These observations underscore the significance of fat distribution and lipid dysregulation in respiratory health. To delve deeper into this association, our study integrated a machine-learning approach to assess the collective impact of lipid-related parameters on pulmonary function.

While the precise mechanisms through which AIP impacts pulmonary function remain unclear, emerging evidence links this pathway to lipid metabolism disorders. Dysregulation in lipid balance modifies the cellular environment within the alveoli, leading to chronic inflammation and mechanical constraints that diminish FVC and FEV_1_ (26–29). The accumulation of thoraco-abdominal fat, which includes diaphragmatic displacement caused by abdominal adiposity, perturbs thoracic mechanics, resulting in reduced lung volume and functional residual capacity, while compromising chest wall and pulmonary compliance (30–32). Obese individuals also exhibit increased airway resistance (33, 34), a phenomenon exacerbated by adipokines such as leptin, TNF-α, IL-6, lipocalin, and C-reactive protein (35). These mediators induce direct airway inflammation and promote immune-mediated structural remodeling (36, 37), culminating in persistent low-grade inflammation that drives airway fibrosis (38), diminished lung compliance, and ventilatory dysfunction (39, 40). Moreover, the mechanisms underlying gender disparities in the association between pulmonary function and AIP remain inadequately understood. Several studies indicate that women typically exhibit elevated levels of total cholesterol and HDL-C compared to men, while LDL-C and triglyceride concentrations are comparable between genders (41). Notably, women exhibit a higher proportion of large HDL particles, constituting 65% of total HDL (45% in men), and have fewer small HDL particles (42). Given the anti-atherosclerotic properties associated with large HDL particles, this difference may elucidate the comparatively lower cardiovascular risk observed in women, even when lipid levels are similar. Our analysis using a threshold effect approach revealed a U-shaped relationship between AIP and pulmonary function (FEV_1_, FVC) in females but not in males. This non-linear association suggests that in women, moderate increases in AIP may be linked to decreased lung function, while beyond a certain threshold, the association plateaus or even reverses. This non-linear and gender-specific trend might be explained by a number of biological processes. Estrogen is essential for controlling inflammation and lipid metabolism. By augmenting the proportion of large HDL particles with potent anti-inflammatory and antioxidant properties, estrogen may mitigate pulmonary impairment induced by AIP (43). Smaller HDL particles, on the other hand, are associated with pro-inflammatory conditions and offer diminished protection when estrogen levels decline post-menopause. Additionally, estrogen influences pulmonary immune responses by impacting cytokine production and immune cell activation. Studies have indicated that estrogen can exacerbate lung inflammation by modulating cytokine generation. For instance, estrogen has been shown to stimulate the production of pro-inflammatory cytokines in the lungs, such as TNF-α and IL-8, thereby heightening inflammatory reactions (44, 45). Interestingly, the observed U-shaped correlation may also reflect compensatory or treatment-related behaviors. Even in cases where overall lipid levels appear satisfactory, alterations in lipid quality (such as smaller HDL particles) may still contribute to lung damage at lower AIP levels. Conversely, individuals with higher AIP levels may have initiated lipid-lowering interventions or exhibit adaptive antioxidant responses that mitigate further lung damage. High AIP may also trigger systemic or hepatic stress responses that lower systemic inflammation or encourage lipid balance (46). These combined mechanisms may alleviate the adverse impact of high AIP levels on lung function at the extreme end. The sex-specific variations in immune responses, hormonal regulation, and lipid profiles could underlie the non-linear association between AIP and pulmonary function in females. The interaction of estrogen, inflammation, HDL particle composition, and potential threshold effects underscores the critical importance of considering sex as a biological variable in studies investigating pulmonary outcomes associated with lipids. Furthermore, neither PEF nor the FEV_1_/FVC ratio exhibited a significant correlation with AIP. These parameters exert distinct physiological effects, which might explain this discrepancy. PEF is subject to variables unrelated to lipid metabolism, primarily reflecting the peak expiratory flow rate determined largely by airway caliber, expiratory muscle effort, and neuromuscular coordination (47, 48). Conversely, the FEV_1_/FVC ratio may lack sensitivity to early alterations in lung function linked to atherogenic dyslipidemia and is primarily employed for detecting obstructive ventilatory impairments (49). These variations imply that rather than consistently affecting all spirometric indices, AIP may preferentially impact specific elements of lung function, such as FEV_1_ and FVC. The findings enhance the understanding of the relationship between lipid metabolism and pulmonary well-being by demonstrating a significant association between AIP levels and lung function. According to these results, AIP could be a potential biomarker linked to decreased lung function, warranting further investigation in longitudinal studies to assess its utility in clinical risk assessment for lung disorders. It is important to acknowledge several limitations of this study. The cross-sectional nature of the NHANES data impedes the establishment of causal relationships and determination of the precise timing of AIP and lung function deterioration. Although correlations were identified, the directionality of the relationship between increased AIP and pulmonary impairment remains unclear. Further longitudinal or interventional investigations are warranted to validate these findings and unravel the underlying causal pathways. Despite extensive adjustment for potential confounders, residual confounding cannot be fully excluded. Unmeasured significant factors, such as physical activity, dietary quality, and medication usage (e.g., bronchodilators, statins), were not accounted for in the dataset. These variables could affect pulmonary function and AIP, potentially introducing bias to the results. Furthermore, the under-representation of younger individuals limits the study to evaluate relationships between AIP and lung function in young people, primarily due to the scarcity and suboptimal quality of spirometry data within this demographic. This age limitation may affect the external validity and generalizability of our findings to broader populations. Despite these limitations, the study has several notable strengths. The large sample size and nationally representative cross-sectional design enabled modeling of multiple confounders, yielding more robust results. Additionally, subgroup analyses across multiple parameters evaluated the strength of associations between AIP and lung function in different populations.

## 5 Conclusion

The findings of this research indicate a negative correlation between AIP and lung function in individuals in the United States, indicating that AIP may serve as a significant monitoring indication for lung function. However, to substantiate these results, further comprehensive longitudinal investigations are required for further confirmation.

## Data Availability

The original contributions presented in the study are included in the article/Supplementary material, further inquiries can be directed to the corresponding authors.

## Footnotes

**footnote :** ^1^ http://www.r-project.org

**footnote :** ^1^ http://www.empowerstats.com
