# Supplementary material for: The association between high atherogenic index of plasma and impaired lung function: a population-based study
Source: Front Med (Lausanne). 2025 Jul 9;12:1589605. doi: 10.3389/fmed.2025.1589605 (PMC12283284; doi:10.3389/fmed.2025.1589605)
Supplement: Supplementary file 1 [file Supplementary_file_1.docx]

**Supplementary Table S1 Detailed information on categorical covariables**

| **Covariable** | **Range** | | | **Grouping for adjustment** | |  |
| --- | --- | --- | --- | --- | --- | --- |
| **Race** | Mexican American | | | Mexican American | |  |
|  | Non-Hispanic White | | | Non-Hispanic White | |  |
|  | Non-Hispanic Black | | | Non-Hispanic Black | |  |
|  | Other Hispanic | | | Other Hispanic | |  |
|  | Other Race - Including Multi-Racial | | | Other Race | |  |
| **Education level** | Less than 9th grade | | | <High school | |  |
|  | 9-11th grade (Includes 12th grade with no diploma) | | | <High school | |  |
|  | High school graduate/GED or equivalent | | | High school or GED | |  |
|  | Some college or AA degree | | | ≥High school | |  |
|  | College graduate or above | | | ≥High school | |  |
| **Alcohol intake** | had <12 drinks in 1 year | | | No | |  |
|  | had ≥12 drinks in 1 year | | | Yes | |  |
|  | ≥3 drinks per day for females or ≥ 4drinks per day for males | | | Yes | |  |
|  | | |  |  |  |  |
| **Smoke status** | | Smoked less than 100 cigarettes in life | | | No | |
|  | | Smoked at least 100 cigarettes in life | | | Yes | |
| **Diabetes** | NA | | | (1) self-reported diagnosis by a physician or healthcare professional | |  |
|  |  | | | (2) HbA1c (glycated hemoglobin) level over 6.5%, | |  |
|  |  | | | (3) fasting blood glucose (FPG) level over 126 mg/dL (7.0 mmol/L) | |  |
| **Hypertension** | NA | | | (1) Self-reported diagnosis of hypertension | |  |
|  |  | | | (2) BP≥130 mmHg and/or DBP≥80 mmHg | |  |
|  |  | | | (3) the use of antihypertensive medication | |  |
| **Body mass index (kg/m^2^)** | <18.5 | | | Underweight | |  |
|  | 18.5-25 | | | Normal | |  |
|  | 25-30 | | | Overweight | |  |
|  | ≥30 | | | Obese | |  |

**Supplementary Table 2: Sensitivity analysis among lung function paraments and AIP**

| **Lung function** | **AIP** | **Model 1** | | **Model 2** | | **Model 3** | |
| --- | --- | --- | --- | --- | --- | --- | --- |
|  |  | **β（95%CI）** | **p-value** | **β（95%CI）** | **p-value** | **β（95%CI）** | **p-value** |
| FEV₁（ml） |  |  |  |  |  |  |  |
|  | Continuous | 197.11 (98.77, 295.44) | <0.001 | -220.12 (-284.31, -155.92) | <0.001 | -168.77 (-238.44, -99.09) | <0.001 |
|  | Q1(-0.79-0.08) | Reference（0） |  | Reference（0） |  | Reference（0） |  |
|  | Q2(0.08-0.28) | 60.49 (-23.79, 144.78) | 0.160 | -84.43 (-137.27, -31.59) | 0.002 | -62.63 (-114.88, -10.38) | 0.019 |
|  | Q3(0.28-0.50) | 180.11 (95.28, 264.95) | <0.001 | -62.95 (-116.77, -9.13) | 0.022 | -30.85 (-85.71, 24.02) | 0.271 |
|  | Q4(0.50-2.06) | 137.07 (51.04, 223.09) | 0.002 | -204.26 (-260.08, -148.45) | ＜0.001 | -159.10 (-218.42, -99.79) | ＜0.001 |
|  | p for trend | <0.001 |  | ＜0.001 |  | ＜0.001 |  |
| FVC（ml） |  |  |  |  |  |  |  |
|  | Continuous | 359.55 (238.21, 480.89) | <0.001 | -231.79 (-312.25, -151.32) | <0.001 | -171.89 (-259.63, -84.15) | <0.001 |
|  | Q1(-0.79-0.08) | Reference（0） |  | Reference（0） |  | Reference（0） |  |
|  | Q2(0.08-0.28) | 128.31 (24.30, 232.32) | 0.016 | -79.41 (-145.69, -13.14) | 0.019 | -52.58 (-118.42, 13.26) | 0.118 |
|  | Q3(0.28-0.50) | 280.49 (175.80, 385.18) | <0.001 | -57.69 (-125.19, 9.82) | 0.094 | -19.29 (-88.42, 49.85) | 0.585 |
|  | Q4(0.50-2.06) | 279.22 (173.06, 385.38) | ＜0.001 | -208.18 (-278.19, -138.18) | ＜0.001 | -156.37 (-231.11, -81.63) | ＜0.001 |
|  | p for trend | ＜0.001 |  | ＜0.001 |  | ＜0.001 |  |
| FEV₁/FVC |  |  |  |  |  |  |  |
|  | Continuous | -0.02 (-0.03, -0.01) | <0.001 | -0.01 (-0.02, -0.00) | 0.0103 | -0.01 (-0.02, -0.00) | 0.0320 |
|  | Q1(-0.79-0.08) | Reference（0） |  | Reference（0） |  | Reference（0） |  |
|  | Q2(0.08-0.28) | -0.01 (-0.02, -0.00) | 0.006 | -0.01 (-0.01, 0.00) | 0.060 | -0.01 (-0.01, 0.00) | 0.065 |
|  | Q3(0.28-0.50) | -0.01 (-0.02, -0.00) | 0.009 | -0.00 (-0.01, 0.00) | 0.141 | -0.00 (-0.01, 0.00) | 0.189 |
|  | Q4(0.50-2.06) | -0.02 (-0.03, -0.01) | ＜0.001 | -0.01 (-0.02, -0.00) | 0.002 | -0.01 (-0.02, -0.00) | 0.007 |
|  | p for trend | ＜0.001 |  | 0.004 |  | 0.015 |  |
| PEF (ml/s) |  |  |  |  |  |  |  |
|  | Continuous | 871.37 (631.03, 1111.70) | ＜0.001 | -233.73 (-416.91, -50.56) | 0.0124 | -227.76 (-424.50, -31.03) | 0.0233 |
|  | Q1(-0.79-0.08) | Reference（0） |  | Reference（0） |  | Reference（0） |  |
|  | Q2(0.08-0.28) | 294.15 (87.84, 500.46) | 0.005 | -66.65 (-217.54, 84.24) | 0.387 | -64.29 (-211.98, 83.41) | 0.394 |
|  | Q3(0.28-0.50) | 563.97 (356.31, 771.63) | ＜0.001 | -66.64 (-220.32, 87.05) | 0.396 | -73.70 (-228.80, 81.39) | 0.352 |
|  | Q4(0.50-2.06) | 661.03 (450.46, 871.60) | ＜0.001 | -247.34 (-406.72, -87.96) | 0.002 | -248.60 (-416.27, -80.94) | 0.004 |
|  | p for trend | ＜0.001 |  | 0.003 |  | 0.005 |  |
| FEF25-75%(ml/s) |  |  |  |  |  |  |  |
|  | Continuous | 69.96 (-75.21, 215.14) | 0.3450 | -213.67 (-331.80, -95.55) | ＜0.001 | -238.10 (-367.91, -108.29) | ＜0.001 |
|  | Q1(-0.79-0.08) | Reference（0） |  | Reference（0） |  | Reference（0） |  |
|  | Q2(0.08-0.28) | 13.45 (-110.90, 137.80) | 0.832 | -79.88 (-177.07, 17.31) | 0.107 | -84.42 (-181.78, 12.94) | 0.089 |
|  | Q3(0.28-0.50) | 168.51 (43.35, 293.68) | 0.008 | -8.14 (-107.13, 90.85) | 0.872 | -19.33 (-121.56, 82.91) | 0.711 |
|  | Q4(0.50-2.06) | 5.91 (-121.01, 132.83) | 0.927 | -219.76 (-322.42, -117.10) | ＜0.001 | -233.13 (-343.66, -122.61) | ＜0.001 |
|  | p for trend | 0.476 |  | ＜0.001 |  | ＜0.001 |  |

Model 1 was not modified. Age, sex, and race were adjusted for Model 2. Every variable in Model 3 has been adjusted.
